# Development of SAFT-Based Coarse-Grained Models of Carbon Dioxide and Nitrogen

**DOI:** 10.1021/acs.jpcb.5c00536

**Published:** 2025-03-21

**Authors:** Alexandros Chremos, William P. Krekelberg, Harold W. Hatch, Daniel W. Siderius, Nathan A. Mahynski, Vincent K. Shen

**Affiliations:** Chemical Sciences Division, National Institute of Standards and Technology, Gaithersburg, Maryland 20899-8320, United States

## Abstract

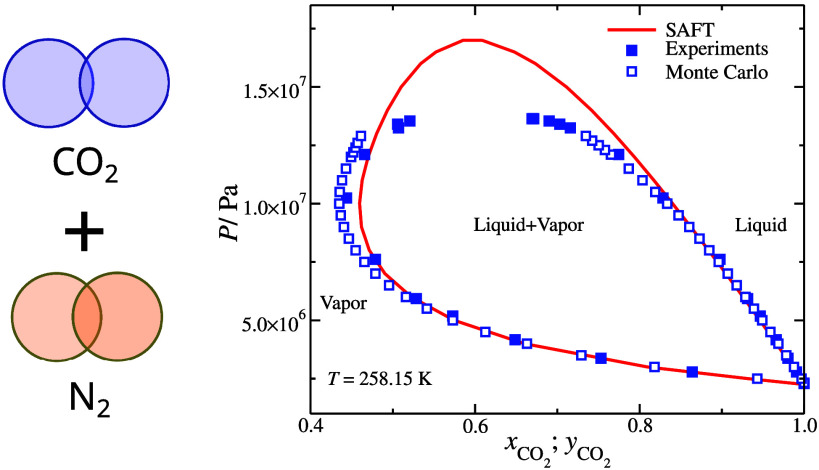

We develop coarse-grained models for carbon dioxide (CO_2_) and nitrogen (N_2_) that capture the vapor–liquid equilibria of both their single components and their binary mixtures over a wide range of temperatures and pressures. To achieve this, we used an equation of state (EoS), namely Statistical Associating Fluid Theory (SAFT), which utilizes a molecular-based algebraic description of the free energy of chain fluids. This significantly accelerates the exploration of the parameter space, enabling the development of coarse-grained models that provide an optimal description of the macroscopic experimental data. SAFT creates models of fluids by chaining together spheres, which represent coarse-grained parts of a molecule. The result is a series of fitted parameters, such as bead size, bond length, and interaction strengths, that seem amenable to molecular simulation. However, only a limited set of models can be directly implemented in a particle-based simulation; this is predominantly due to how SAFT handles overlap between bonded monomers with parameters that do not translate to physical features, such as bond length. To translate such parameters to bond lengths in a coarse-grained force-field, we performed Wang–Landau transition-matrix Monte Carlo (WL-TMMC) simulations in the grand canonical ensemble on homonuclear fused two-segment Mie models and evaluated the phase behavior at different bond lengths. In the spirit of the law of corresponding states, we found that a force field, which matches SAFT predictions, can be derived by rescaling length and energy scales based on ratios of critical point properties of simulations and experiments. The phase behavior of CO_2_ and N_2_ mixtures was also investigated. Overall, we found excellent agreement over a wide range of temperatures and pressures in pure components and mixtures, similar to TraPPE CO_2_ and N_2_ models. Our proposed approach is the first step to establishing a more robust bridge between SAFT and molecular simulation modeling.

## 1 Introduction

Carbon dioxide (CO_2_) plays a vital role in ecosystems via a circular journey through a diverse range of biological and physical processes contributing to the existence of life. For example, CO_2_ is absorbed by plants during photosynthesis and then stored in roots, which in turn is released when the plants decay.^1^ Some of this carbon becomes trapped in rocks and other geological deposits under the Earth’s surface, forming coal and other fossil fuels after millions of years.^2^ However, there are growing concerns that the impact of anthropogenic emissions, by extraction and consumption of fossil fuels, may cause disruptions in natural-based carbon circulations, causing irreversible damage to these ecosystems.^3,4^ A notable example is in the marine food chains, which are threatened by ocean acidification^5^ caused by absorbing increased quantities of CO_2_ from the atmosphere.^6^

Inspired by the circular processes found in nature, a new kind of industrial framework, namely, circular economy, is emerging.^7,8^ The aim of this framework is to reduce anthropogenic emissions with processes that keep target materials in closed-loop processes. The rise of this concept in recent years has placed CO_2_ at center stage as a vital component in what has been termed the “carbon economy”,^9,10^ with several technologies for utilization of the captured carbon in development, e.g., as a material component for plastics,^11^ jet-fuel^12^ and biofuel,^13^ and other useful chemicals.^14^ One way to remove CO_2_ from the atmosphere is with direct air capture (DAC);^15,16^ alternatively, carbon capture can take place in large-scale industrial or power-production processes, reducing CO_2_ emissions before being released into the atmosphere. Captured CO_2_ is then compressed and transported by means of a pipeline or ship to the desired location, e.g., injected into a geological storage reservoir or to a postprocessing facility. However, the stream exported from the capture plant may contain many impurities, such as nitrogen (N_2_), hydrogen (H_2_), oxygen (O_2_), sulfur dioxide (SO_2_), nitrogen oxides (NO_*x*_), or water (H_2_O).^17^

The thermophysical properties of CO_2_ and its mixtures with impurities and reservoir fluids (brines and hydrocarbons) considerably impact the design and operation of the transportation and storage processes. For instance, the optimal stream transfer through a pipeline requires the suppression of any phase separation of impure CO_2_.^18,19^ Moreover, CO_2_ storage capacity of a saline aquifer is determined by the saturated phase densities and interfacial properties of the (CO_2_ + impurities + brine) system under reservoir conditions.^20,21^ Hence, to improve the process design and simulation of these systems, accurate thermodynamically consistent molecular models need to be developed based on phase equilibrium and thermophysical data for CO_2_-rich mixtures.

In the current contribution, we take a step toward harmonizing molecular simulations and equations of state for these complex mixtures. The equation of state (EoS) we use is the statistical associating fluid theory (SAFT),^22,23^ which is based on the first-order thermodynamic perturbation theory (TPT1) of Wertheim.^24−28^ The underlying model is a chain of spherical monomers that interact through a particular potential; in this work, we choose to utilize the Mie potential. Different chain models are more appropriate for different types of chain fluids, for example, SAFT-VR Mie is applicable for homonuclear chains,^29^ while SAFT-γ Mie is required for heteronuclear ones.^30^ SAFT has been frequently used to model pure components and mixtures relevant to carbon capture applications.^31−33^ We note that there are numerous theories and models described in the literature that can address some of the challenges mentioned above; notable examples include eNRTL theory^34,35^ and UNIQUAC,^36−39^ which has been extended to study the absorption of CO_2_ in aqueous solutions of alkanolamines.^40,41^ A review of these theories and models is out of the scope of the current contribution, and we direct the reader to refs (42−45). Unlike these methods, SAFT is suitable for our study because it provides the Helmholtz free energy in a closed algebraic form given explicitly in terms of a predefined intermolecular potential and a molecular model suitable for molecular simulations. Indeed, molecular simulations are necessary to obtain material properties beyond what an EoS can provide, such as structural information or dynamics. The advantage of using an EoS of this type is the tremendous increase in speed at which we can explore a wide parameter space to obtain an optimal description of the macroscopic experimental data. There are SAFT-γ Mie databases^46,47^ that contain highly optimized parameter sets fitted to experiments over a wide range of conditions. However, the majority of these parameter sets cannot be transferred to molecular simulations due to the approach used in SAFT EoS in modeling fused (overlapping) chain fluids. Instead, new models need to be developed utilizing a subset of SAFT’s formulation,^48,49^ meaning that a bridge between SAFT and molecular simulations remains unrealized.

Molecular simulations are an alternative approach to experimental measurements and theoretical techniques for obtaining fluid and material properties. By using a microscopic representation, i.e., a molecular model along with a functional form to approximate the intra- and intermolecular forces, one can simulate the state of matter of a particular substance at given thermodynamic conditions by either Monte Carlo (MC) or molecular dynamics (MD) methods.^50,51^ Simulations of vapor–liquid equilibrium (VLE) with either method often encompass the interface between these two phases, which increases in length scale near the critical point requiring large-scale simulations that were slow to equilibrate. Tuning molecular models to describe the VLE was a considerable obstacle in the 1980s and 1990s, and one that remains a contemporary challenge if constrained by computational resources. Panagiotopoulos spearheaded the development of the Gibbs Ensemble Monte Carlo technique, which enables the simulation of these systems without an interface, thus overcoming the issues occurring in MD.^52−54^ This innovative approach inspired other researchers to expand upon this idea and numerous novel techniques have been developed since then, such as multicanonical,^55,56^ transition-matrix,^57−59^ and Wang–Landau algorithms.^60,61^ MC approaches are an invaluable tool for probing the phase behavior of molecular models in single components and their mixtures; examples in the study of CO_2_ and its mixtures include but are not limited to refs (62−66). In our study, we will utilize approaches inspired by Panagiotopoulos and other scholars to develop coarse-graining (CG) models for substances of interest resulting in the beginnings of a bridge between SAFT and molecular simulations.

The underlying motivation for our work is to strengthen the connection between SAFT and coarse-graining (CG) models, by bringing the high-fidelity global representation of SAFT into CG molecular modeling process. In particular, our current study aims to develop CG models for CO_2_ and N_2_ by mapping the predictions of SAFT-γ Mie EoS to molecular simulation models. We performed Wang–Landau transition-matrix Monte Carlo (WL-TMMC) simulations of a two-segment Mie model (similar to its representation in the SAFT model) in the grand canonical ensemble and evaluated the phase behavior of this model with a variation of the bond length of the fused spheres. Based on our findings, we propose scaling relations to readjust the energy and length units based on the ratio of the molecular simulations and experimental critical points. Our proposed approach enables SAFT-γ Mie parameters to be transferred to molecular simulations of pure components and their mixtures.

Our paper is organized as follows. Section 2 contains details of the model and simulation methods. The development of the coarse-grained models and the results are presented in Section 3. We conclude in Section 4.

## 2 Model and Methodology

The coarse-graining strategy used in our work is based on the assumption that SAFT models (homonuclear or even heteronuclear fused Mie monomers) can model a wide range of real substances. However, only SAFT fused chain models are utilized to develop CG models for simulations,^48,49^ as discussed in Section 1. This limitation of the SAFT EoS will be further discussed below, but remedying this is the main goal of our work. Here, we outline the model and methods used in the current study.

### 2.I Molecular Model

**Figure 1:**
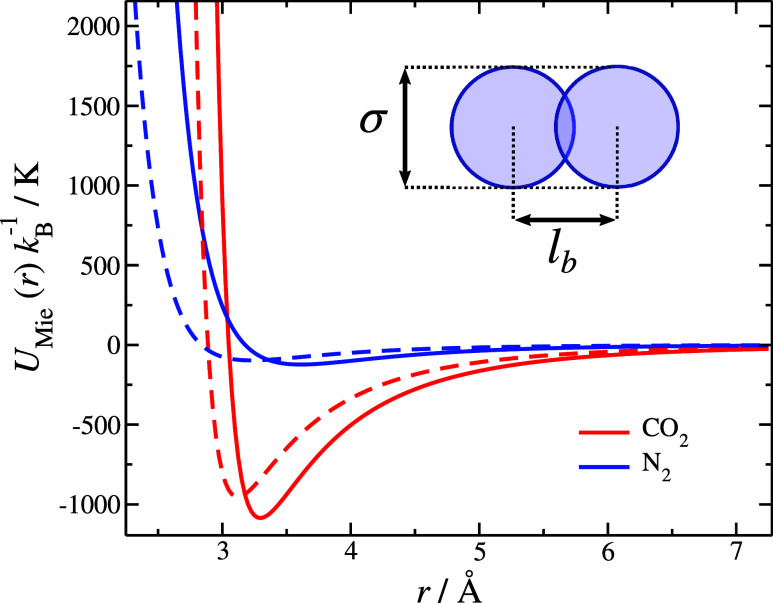
Interaction potentials for the monomers composing CO_2_ and N_2_ based on the parameter sets obtained from SAFT (continuous lines) and the rescaled parameters for molecular simulations (dashed lines); the parameter sets for each case are presented in Table 1. A schematic of the coarse-grained fused two-segment model used in the simulations is also presented.

The models of CO_2_ and N_2_ are based on a chain having *n* = 2 identical Mie monomers, which are slightly fused (overlapping) with a bond length, *l*_b_, see inset of Figure 1. For our current study, we consider the bonds to be rigid.

The Mie potential^67^ acting between two monomers is expressed as,
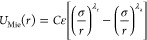
1where *r* is the distance between two Mie monomers, *ε* is the potential depth, *σ* is the position at which the potential is zero (monomer diameter), and *λ*_r_ and *λ*_a_ are the repulsive and attractive exponents which characterize the pair potential energy, respectively. The constant *C* ensures that the minimum of the potential corresponds to −*ε* and it is defined as,
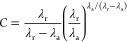
2Several studies have focused on the phase behavior of the Mie potential.^68^

For mixtures, we utilize the following mixing rules for the Mie potentials to determine the cross-interaction parameters of two monomers *i* and *j*,^29,30^
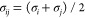
3
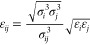
4
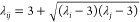
5unless stated otherwise.

### 2.II SAFT

The general SAFT form of the Helmholtz free energy of a chain fluid composed of Mie monomers can be written as,
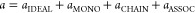
6where *a* = *A*/(*Nk*_B_*T*) is the dimensionless Helmholtz free energy, with *k*_B_ is the Boltzmann constant, *T* is temperature, and *N* is the number of molecules. In eq 6, *a*_IDEAL_ is the free energy of the ideal particle, *a*_MONO_ is the residual free energy due to the (nonbonded) monomers, *a*_CHAIN_ is the contribution due to the formation of chains composed of monomers, and *a*_ASSOC_ is the contribution of anisotropic interactions. For more details on SAFT-VR Mie and SAFT-γ Mie see the refs (29,30), respectively. Note that for the current study, the last term is not utilized since no hydrogen bonds are present in our systems, and so *a*_ASSOC_ = 0; for the development of the association term, see ref (69).

All SAFT calculations were performed with Despasito,^70^ which is an open-source application for thermodynamic calculations and parameter fitting with SAFT. The SAFT parameters used in our current study for CO_2_ and N_2_ are presented in Table 1.

**Table 1:** SAFT-γ Mie and CG Self- and Cross-Interaction Model Parameters for CO_2_ and N_2_ Functional Groups^a^

		SAFT-γ Mie parameters					CG model parameters^b^					
group(s)	*n*	*S*	*σ*/Å	(*ε*/*k*_B_)/K	*λ*_r_	*λ*_a_	references	*l*_b_/σ	*σ*/Å	(*ε*/*k*_B_)/K	*λ*_r_	*λ*_a_
CO_2_	2	0.8468	3.050	207.89	26.408	5.055	(71)	0.9	2.8882	182.364	26.408	5.055
N_2_	2	0.7101	3.178	73.640	10.109	6.000	(72)	0.7	2.9550	49.590	10.109	6.000
CO_2_ + N_2_			3.114	151.90	20.270	5.483	(72)		2.9216	121.500	20.270	5.483

a The SAFT-γ Mie parameters are to be used in the SAFT framework, while the CG model parameters are to be used in molecular simulations.

b Current work.

It should be noted that in the optimization of SAFT parameters, typically *λ*_a_ is kept fixed *λ*_a_ = 6, as this represents the dipole–dipole interactions at long-range distances. For molecules known to deviate from this behavior, e.g., having a quadrupole, then *λ*_a_ becomes a variable, as in the case of CO_2_. In the SAFT Mie theory, the single segment representation of CO_2_, *λ*_a_ = 6.66,^63^ and in the SAFT two-segment representation, *λ*_a_ = 5.055.^71^ Thus, the SAFT models implicitly incorporate some information on the underlying molecular structure.

### 2.III Simulation Methods, Single Component

All molecular simulations used the open-source FEASST version 0.25.1 simulation software.^73^ The simulations utilized the Grand Canonical Transition-Matrix Monte Carlo (GC-TMMC) technique,^74−76^ with Wang–Landau initialization.^77,78^ The key output of these simulations is the *macrostate probability distribution*, Π(**N**; **μ**, *V*, *T*), which quantifies the probability of observing a certain number of particles **N**, the imposed chemical potential(s) **μ**, system volume *V*, and temperature *T*.^74^ Vapor–liquid equilibrium conditions may be identified by histogram reweighting Π(**N**; **μ**, *V*, *T*) until the conditions of phase equilibrium (equality of phase pressures for a given μ and *T*) are satisfied.^74^

For the pure species, the MC simulations of dimers with fixed bond length were performed in cubic simulation boxes of length *L*/*σ* = 10 at several temperatures, with all units reduced by σ and ε.^50^ The vapor–liquid phase diagram of each species was assembled from the simulations in reduced units, after which values of σ and ε were assigned by fitting the phase diagram to NIST correlations^79^ (see Section 2.V). We note that the NIST correlations are based on an empirical equation of state for carbon dioxide^80^ and nitrogen.^81^ Analytic long-range corrections for interactions beyond cutoff distances of *r*_c_/*σ* = 3 were applied by assuming a pair distribution of unity.^82^

Single-molecule translations or rotations were attempted with equal probability using maximum displacement parameters tuned for 25% acceptance in every 10^6^ trials during Wang–Landau equilibration.^83^ Single-molecule insertions and deletions, attempted with equal probability, were twice as likely to be attempted as translations and rotations. Single component simulations were run for up to 2 days parallelized over 32 cores^84^ on dual Intel Xeon Silver 4216 processors with 2.1 GHz base frequency, resulting in at least 1.5 × 10^9^ MC trials during the transition matrix production phase for each processor.

The critical temperature *T*_c_ was estimated using the Wegner expansion up to the first-order correction term,^85,86^
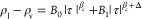
7where *ρ*_l_ and *ρ*_v_ correspond to the coexisting liquid and vapor densities, respectively, *τ* = 1 – *T*/*T*_c_, *β*_c_ = 0.325 is the critical exponent that is fixed at its universal renormalization-group value, Δ = 0.5 is the gap exponent, and *B*_*i*_ are the correlation amplitudes. The critical density *ρ*_c_ is calculated by means of a least-squares fit of the rectilinear diameter law,
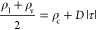
8where *D* is the correlation parameter. The critical pressure *P*_c_ is obtained by extrapolation using the Clausius–Clapeyron equation,^87−89^
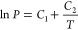
9where *C*_1_ and *C*_2_ are correlation parameters.

### 2.IV Simulation Methods, Binary Mixtures

Following the assignment of *σ* and *ε* for N_2_ and CO_2_, we performed simulations of mixtures to identify vapor–liquid equilibrium at four temperatures below the critical point of CO_2_. Pair interactions between the unlike segments were initially set using combining rules for Mie potentials^29^ and later adjusted to better match the vapor–liquid equilibria (VLE) curves (see Section 3). TMMC simulations of mixtures of N_2_ and CO_2_ were done in a slightly different manner, using a strategy adapted from GC-TMMC simulations of binary Lennard-Jones fluids by Shen and Errington;^76,90^ we note that the macrostate is defined by (*N*_N_2__, *N*_CO_2__) and Π(**N**; **μ**, *V*, *T*) is a function of the two-dimensional macrostate. In this approach, three types of simulations are run: (1) GC-TMMC of pure N_2_, (2) GC-TMMC pure CO_2_, and (3) semigrand-TMMC of an isochoric mixture of N_2_ and CO_2_ in which the number of N_2_ and CO_2_ molecules fluctuate subject to the total number of particles remaining fixed, i.e., constant *N*_total_ “diagonals” in the *N*_N_2__ – *N*_CO_2__ plane. Simulation types 1 and 2 are responsible for the macrostates with *N*_CO_2__ = 0 and *N*_N_2__ = 0, respectively, and yield information about Π(**N**; **μ**, *V*, *T*) along those macrostate axes. Type 3 simulations are run for values of *N*_total_ = *N*_N_2__ + *N*_CO_2__ and yield Π(*N*_N_2__; *N*_total_, μ_N_2__, μ_CO_2__, *V*, *T*). The mixture simulations do not use conventional GC insertion and deletion moves, but instead employ identity-change moves in which an N_2_ molecule is transformed into a CO_2_ molecule (and vice versa), with acceptance criteria based on a difference in imposed chemical potential between the two species.^91^ The full Π(**N**; **μ**, *V*, *T*) is recovered by combining the results of the two pure-species simulations with the set of diagonal simulations. The simulations used to compute the binary Π(**N**; **μ**, *V*, *T*) used cubic boxes of length *L* = 30 Å with segment–segment interactions truncated at a cutoff radius of 9 Å. Analytic long-range corrections were applied as in the single-component simulations. We note that this box size and cutoff radius are slightly larger than those of the single-component simulations based on the *σ* and *ε* that were ultimately assigned to N_2_ and CO_2_ (see Table 1). Simulations of types 1 and 2 used the same move types and tuning strategies as the previous single-component simulations, with selection probability ratios 0.2:0.2:0.6 for translation/rotation/transfer moves. Type 3 simulations used translations, rotations, and identity-change moves with equal probability. All simulations were run until 200 TMMC sweeps^92^ were completed; this corresponds to at least 250 × 10^6^ trial moves for type 3 simulations and 450 × 10^6^ trial moves for type 1 and 2 simulations. The runtime of individual simulations varied dramatically due to the wide range of densities that must be simulated; however, it was generally possible to complete all simulations at one temperature in 80 wallclock hours using six workstations with 32 CPU cores of the type described above per workstation.

### 2.V Model Evaluation

The temperature range of the vapor–liquid equilibrium data considered for evaluation of the coarse-grained models in our current study was between the triple point and 0.9 *T*_c_^exp^, where *T*_c_^exp^ is the experimental critical temperature of the substance under study. Temperatures closer to the critical point were not considered. We note that SAFT is a classical EoS and cannot provide an accurate simultaneous description of the thermodynamic properties both close to and far from the critical region.

To assess the quality of the fluid-phase equilibrium data obtained with the optimal parameter values, we used the average absolute deviations (% ADD),
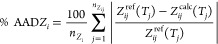
10where *Z*_*i*_ is the vapor pressure or saturated densities of component *i* at data point *j*(*T*_*j*_) and *n*_*Z*_*i*__ is the total number of data points for that component.

## 3 Results and Discussion

There is extensive literature on using SAFT to describe molecular properties over a wide range of fluids.^93^ Several such efforts use SAFT as a framework to generate molecular parameters obtained by fitting experimental results in the EoS framework that are also transferable to molecular simulations.^48,49^ This transferability is feasible, but there are two caveats. The first caveat is that SAFT must be used *without* association sites^94^ and the second caveat is that the chain monomers should not be fused; ignoring these caveats results in deviations between the SAFT predictions and the molecular simulation results. The former is out of the scope of our current study, but we seek to rectify the latter caveat.

SAFT provides an algebraic functional form of the free energy of (associative) chain fluids, which can be used in a general way because it relies on a relatively simple coarse-grained model, i.e., monomers connected with rigid bonds, see eq 6. Specifically, the *a*_CHAIN_ term describes how the overall free energy of a chain fluid changes compared to having an identical fluid without bonds, i.e., *a* = *a*_IDEAL_ + *a*_MONO_. The *a*_CHAIN_ term was originally developed with nonfused monomers in mind, so the number of monomers in the chain was an integer.^23^ The development of SAFT-VR^29,95^ relaxed this constrain to further improve the predictions of the theory with experimental findings, resulting in chain molecules having a noninteger number of monomers, e.g., SAFT-VR SW of N_2_ has 1.33 monomers.^96^

**Figure 2:**
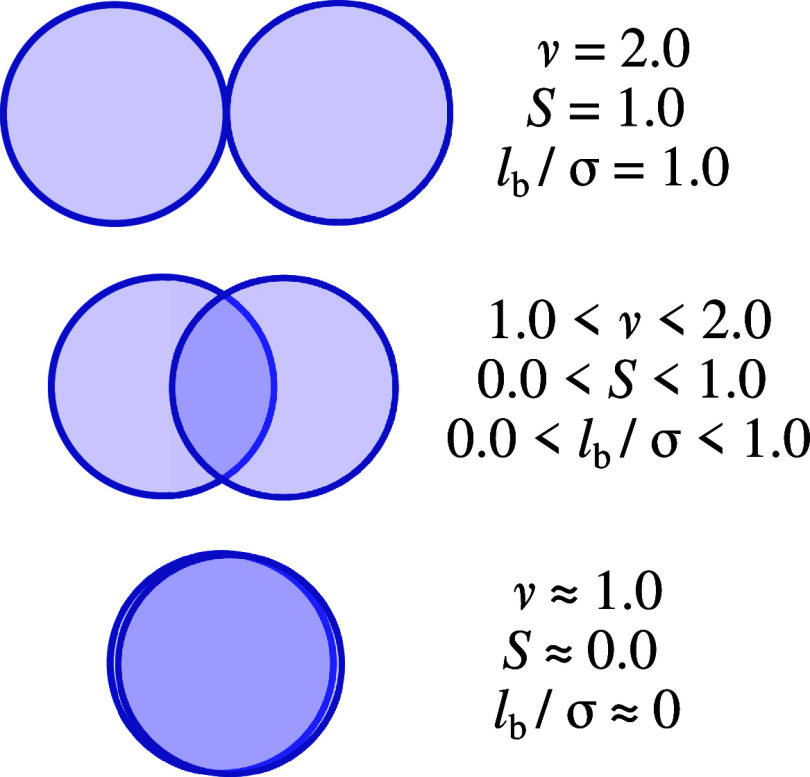
Schematic illustrating the degree of overlap by two identical spheres and the different notations used in SAFT to describe it. In SAFT-VR, the effective number of monomers, i.e., 1.0 < *ν* < 2.0, is used, while in SAFT-γ, the shape factor of 1 of the spheres notation is used, i.e., 0.0 < *S* < 1.0, as discussed in the main text. The bond length utilized in molecular simulations, 0.0 < *l*_b_/*σ* < 1.0 is also presented.

In SAFT-γ (heteronuclear chain model),^30^ instead of using a noninteger number of monomers, a new parameter was introduced called shape factor, *S*, which is assigned to each monomer. It effectively describes the degree of the contribution of a particular monomer to thermodynamic behavior. When the monomer does not overlap with its bonded monomers, then *S* = 1, meaning that the specific monomer fully contributes to thermodynamic behavior, and when it is completely overlapped, then *S* = 0, i.e., it does not contribute to thermodynamic behavior, see Figure 2. For a two-segment structure, the *S* parameter can be qualitatively understood as the degree of overlap of two spheres. However, it becomes challenging for longer chains to decipher the physical meaning of *S* since additional molecular features, such as bond angles and dihedrals, contribute to the chain fluids’ thermodynamics. At this point, it is important to note that, for the systems (homonuclear chains) in our current study, there is no substantive difference between SAFT-VR Mie and SAFT-γ Mie EoSs, since the definition of *S* and noninteger number of monomers are equivalent. Nevertheless, we choose to use the notation SAFT-γ Mie EoS in our paper.

**Figure 3:**
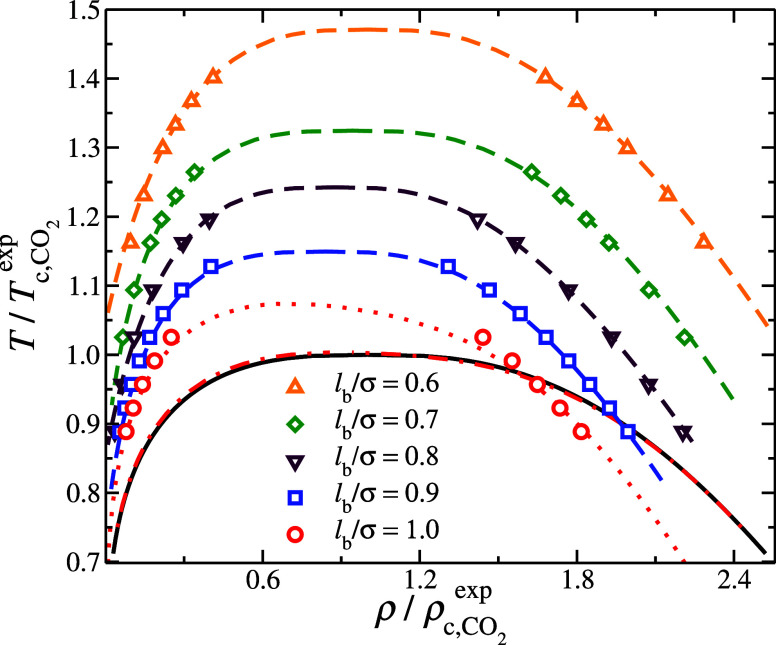
Vapor–liquid equilibria normalized by the experimental estimate of the critical point of CO_2_. The symbols correspond to MC simulations at different bond lengths, the black continuous line corresponds to the NIST correlations for CO_2_,^79^ dashed lines correspond to Wegner correlations, see eqs 7 and 8, and the dotted and dot-dashed lines correspond to SAFT predictions based on the parameter set presented in Table 1 having *S* = 1 and *S* = 0.8468, respectively.

Now that we have provided a brief background on the technical aspects of SAFT models, we will focus on the modeling of CO_2_. There are two published SAFT Mie CO_2_ models. The first one is based on a single segment,^63^ and the second one is based on two fused monomers.^71^ When we evaluated the former, we found that it exhibited a good agreement with the experimental behavior of pure CO_2_, but the performance deteriorated in mixtures (for more details, see Supporting Information). This led us to adopt the two-segment SAFT Mie model.^71^ We briefly note that we encountered the same issue when we attempted to develop a SAFT single-segment model for N_2_ and explored the mixture with CO_2_ (results not shown here). The predicted SAFT parameters for the two-segment model of CO_2_ also exhibit excellent performance in describing the VLE behavior for pure component^71^ and mixtures,^71,72^ see Figure 3 (also discussed in detail below). The transferability of these SAFT parameters to molecular simulations becomes challenging because the monomers are fused since *S* < 1. It is unclear how *S* can be mapped onto physical molecular features, e.g., bond length, spring constant, and for longer chain fluids, the bond angle etc.

**Figure 4:**
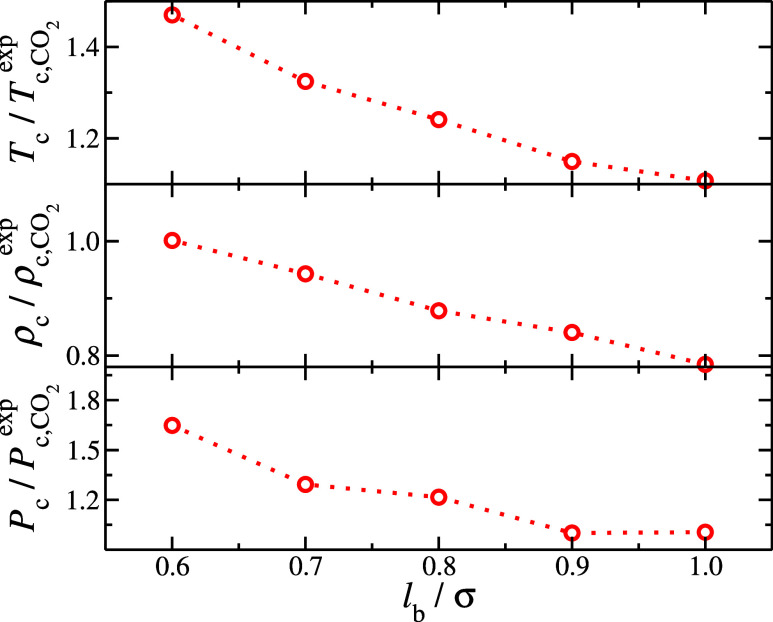
Critical point properties of CO_2_ normalized by the experimental estimates, provided by NIST,^79^ as a function of the bond length, *l*_b_. The critical temperature, *T*_c_, and critical density, *ρ*_c_, were obtained from Wegner correlations, see eqs 7 and 8; the critical pressure, *P*_c_, was calculated with the Clausius–Clapeyron equation, see eq 9.

To better understand this issue, we examine the VLE behavior of a CG model in molecular simulation that is equivalent to the SAFT model used in EoS. In particular, we performed TMMC simulations (see Section 2.III) of two monomers interacting with the Mie potential at different bond lengths, *l*_b_/*σ* = 1.0, 0.9, 0.8, 0.7, and 0.6. The results are presented in Figure 3. The first key observation is that the nonfused CG model, i.e., *l*_b_/*σ* = 1.0, significantly deviates from both the experimental behavior (as described by the NIST correlations) and the SAFT predicted behavior by having a higher *T*_c_ and lower *ρ*_c_, see Figure 3. Moreover, if we use the SAFT parameters presented in Table 1 except that *S* = 1.0 then there is good agreement between the SAFT predictions and the CG model having *l*_b_/*σ* = 1.0. One may anticipate that reducing *l*_b_, i.e., increasing the degree of overlap similar to *S*, would result in the VLE converging toward the predictions of SAFT. However, the observed deviation increases as *l*_b_ decreases, as shown in Figure 4. These results suggest that SAFT-predicted parameters can only be used in molecular simulations if the monomers in the chain model are not fused. Otherwise, the VLE behavior found in simulations is shifted from the SAFT predictions.

We propose to map SAFT’s theoretical results of the fused chain model to molecular simulations. Specifically, we suggest that the parameters obtained from SAFT can be rescaled based on the ratio of critical points obtained from the CG model and the target substance as follows,
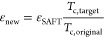
11
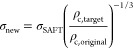
12This type of rescaling is in line with the law of corresponding states, where similar thermodynamic behavior is expected for nonpolar fluids near the critical point.^97^ Similar rescaling approaches are often used to improve the thermodynamic description of a SAFT model.^63,98−100^ The “target” critical point corresponds to the experimental critical point of the substance of interest, and the “original” critical point corresponds to the critical point obtained from the simulation model. The rescaling relations we used effectively replace the units of length and energy predicted by SAFT while keeping the predicted exponents *λ*_r_ and *λ*_a_ the same. Moreover, this rescaling effectively plays the role of *S* in SAFT EoS by readjusting the contribution of the monomers in the simulation since *S* has no direct analog in the simulation model; previous attempts to establish a map of *S* to physical molecular parameters provided adequate performance for dimers^101^ but this was out of the scope of the current study. Once the rescaled parameters are obtained, minor adjustments were performed to further enhance the agreement with target properties. This additional adjustment was necessary since the estimation of the critical point from simulations was not sufficiently accurate; see Section 2.III for more details. We found that the best performance was achieved when *l*_b_/*σ* ≈ 0.9 and *l*_b_/*σ* ≈ 0.7 for CO_2_ and N_2_, respectively; we will use these values of *l*_b_ for the rest of the paper. The rescaled model parameters are presented in Table 1.

**Figure 5:**
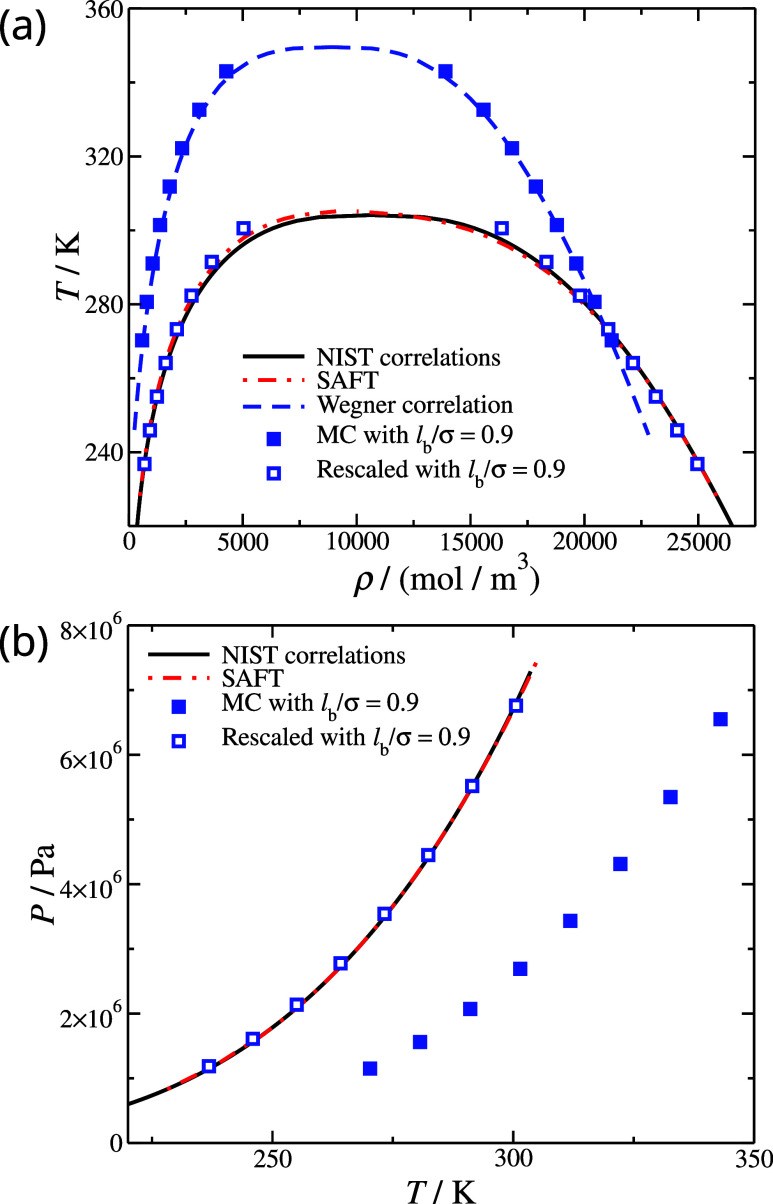
(a) Coexisting densities *Tρ* and (b) vapor pressure *PT* phase diagrams for CO_2_. The black continuous lines correspond to the NIST correlations for CO_2_.^79^ Filled symbols correspond to MC molecular simulations based on SAFT-γ Mie generated parameters, see Table 1; open symbols correspond to the rescaled MC molecular simulation results based on eqs 11 and 12; the rescaled parameters are presented in Table 1.

**Figure 6:**
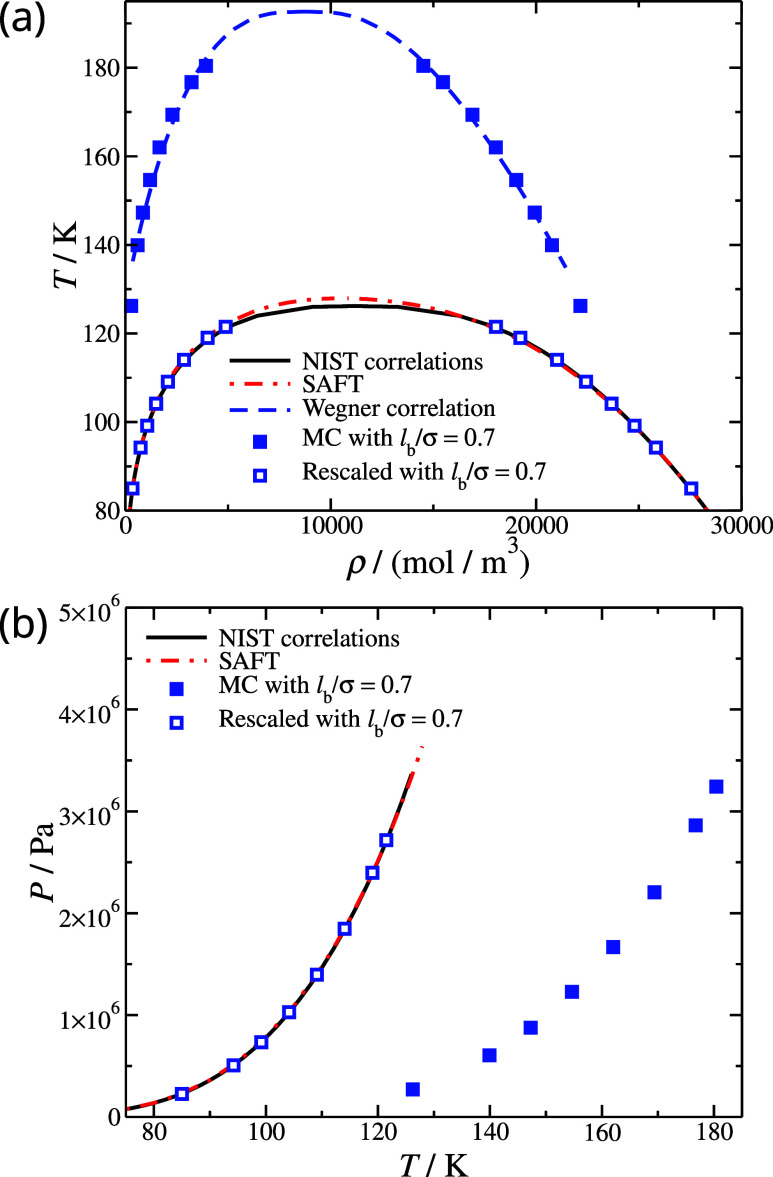
(a) Coexisting densities *Tρ* and (b) vapor pressure *PT* phase diagrams for N_2_. The black continuous lines correspond to the NIST correlations for N_2_.^79^ Filled symbols correspond to MC molecular simulations based on SAFT-γ Mie generated parameters, see Table 1; open symbols correspond to the rescaled MC molecular simulation results based on eqs 11 and 12; the rescaled parameters are presented in Table 1.

We applied these scaling relations to CG models of CO_2_ and N_2_, and the resulting description of the fluid-phase equilibria are seen in Figures 5 and 6, respectively. Moreover, the degree of agreement with reference data is presented in Table 2. Overall, the performance is comparable to frequently used molecular models of CO_2_ and N_2_ used in molecular simulations, such as the TraPPE models.^102^ Nevertheless, we note a couple of additional advantages over these models. First, our CO_2_ model is composed of two monomers rather than three monomers, as in the case of TraPPE, and we do not use partial charges. These differences reduce the computational costs compared to TraPPE models. Second, our models were produced within a single EoS framework, thus ensuring the homogeneity and consistency of the CG models as additional substances are modeled in the future. Indeed, our proposed approach opens a venue for molecular dynamics and Monte Carlo simulations of existing SAFT parameter databases^46,47^ that have been used only within an EoS framework so far. Future work will focus on expanding these scaling relations to chains of *n* > 2 monomers.

**Table 2:** Average Absolute Deviations % ADDs of Vapor Pressure and Saturated Densities for CO_2_ and N_2_ Simulation Models Compared to TraPPE Models^102^^,^^a^

			% ADDs of proposed CG models			% ADDs of TraPPE^102^ models		
component	*T* range/K	*m*	*ρ*_v_	*ρ*_l_	*P*_sat_	*ρ*_v_	*ρ*_l_	*P*_sat_
CO_2_	230–270	5	2.00	0.35	2.69	5.09	0.32	2.19
N_2_	65–105	5	2.46	0.37	1.85	1.28	0.49	0.60

a The results are compared to the NIST correlations^79^ for the particular components by choosing *m* data points equally spaced in the reported temperature range.

**Figure 7:**
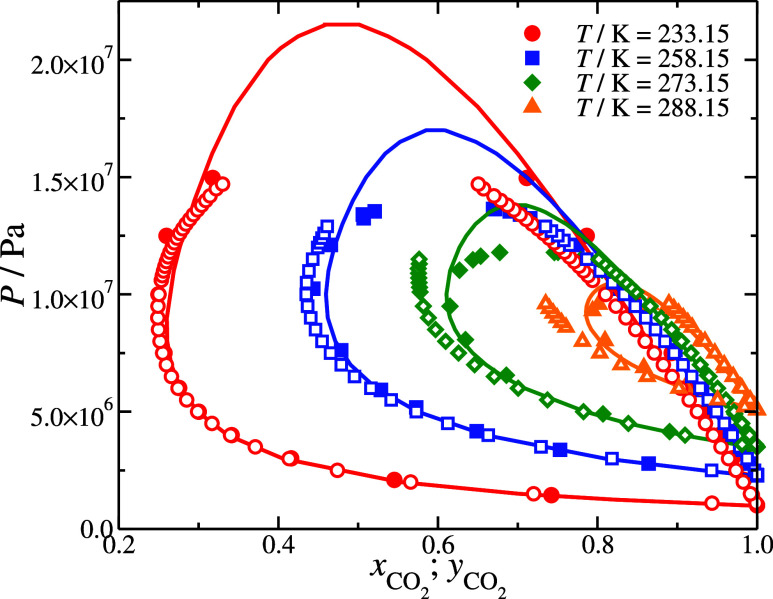
Isothermal pressure–composition slices of the vapor–liquid equilibrium of N_2_ + CO_2_ binary mixture. The continuous lines represent the SAFT-γ Mie predictions based on the parameter set in Table 1. Filled symbols represent experimental bubble and dew point pressures by Fandiño et al.,^103^ and the open symbols correspond to MC simulations based on the parameter set in Table 1.

Now that we have CG molecular parameter sets for CO_2_ and N_2_, which describe the single component VLE behavior, we also examine these models in binary mixtures. We performed TMMC simulations (see Section 2.IV for more details) at *T*/K = 258.15 to estimate the cross energy interaction parameter between CO_2_ and N_2_, *ε*_CO_2_–N_2__ by matching the description of the simulation model with experimental findings. The mixing rules determined all other parameters, see eq 5 and Table 1. Once *ε*_CO_2_–N_2__ was determined, we performed additional MC simulations at *T*/K = 233.15, 273.15, and 288.15 to evaluate the predictions of our models. The resulting phase behavior for the mixtures is presented in Figure 7. In all the temperatures explored, N_2_ was in the supercritical regime, while there was a vapor and a liquid phase for CO_2_.

The critical points are expected to be overestimated in SAFT-γ Mie since it is a classical theory; the development of SAFT-γ Mie parameters of CO_2_ and N_2_ and their mixtures is discussed in refs (71,72). Similarly, we expect our CG models not to adequately describe the critical region since it requires increasing large simulation boxes as the critical point is approached. Thus, deviations are present in the vapor and liquid phases near the critical points. Nevertheless, the simulation data of our CG models and away from the critical point capture the phase behavior of the mixtures as they are in good agreement with the experimental data of Fandiño et al.^103^ The performance is similar to the SAFT-γ Mie predictions, see Figure 7. Based on the reported performance of CO_2_ + N_2_ mixture with TraPPE models, where some deviations in the liquid state were found at lower temperature *T*/K = 253.15,^102^ our CG models provide an improved agreement. This is considerable, given that our CG models do not include partial charges, which significantly increase the computation cost.

## 4 Conclusions

We developed coarse-grained (CG) homonuclear fused two-segment Mie models for carbon dioxide (CO_2_) and nitrogen (N_2_) that capture the vapor–liquid equilibria of their single components and their mixtures over a wide range of temperatures and pressures. To obtain the final models, we utilized an equation of state (EoS), namely Statistical Associating Fluid Theory (SAFT), which provides a molecular-based algebraic description of the free energy, making it a candidate for “bridging” an EoS with coarse-grained models suitable for molecular simulations. This is because some, but not all, parameters in SAFT are interpretable as physical objects in particle-based molecular simulations. Previous studies have used SAFT (and its variants) in two main ways. The first way is by developing databases of functional groups.^46,47^ The second way is developing coarse-grained models based on a subset of SAFT’s formulation, specifically, based on *nonfused* chain models.^48,49^ In other words, SAFT *fused* chain models are not directly transferrable to molecular simulations. To resolve this issue and obtain molecular parameter sets for CO_2_ and N_2_ suitable for molecular simulations, we performed Wang–Landau transition-matrix Monte Carlo (WL-TMMC) simulations in the grand canonical ensemble on homonuclear fused two-segment Mie models and evaluated the phase behavior at different bond lengths. Based on our findings, we propose rescaling the units of energy and length predicted by SAFT-γ Mie by the ratio of critical points obtained from simulations and the experiments. The cross-interaction parameters were also obtained by evaluating the performance of these models in the phase behavior of binary mixtures of CO_2_ and N_2_, and we found an excellent agreement over a wide range of temperatures and pressures. The significance of our approach is that it combines experiments, theory, and molecular simulations,^50^ thus accelerating the development of thermodynamically consistent molecular models and, by extension, material discovery. Our proposed approach is the first step to establishing a more robust bridge between SAFT and molecular simulation modeling.

## Supporting Information Available

Additional details on the WL-TMMC methodology is presented, as well as, additional calculations of the phase behavior of the single-segment model of N_2_ and its performance in binary mixtures with CO_2_ (PDF)
