# Supplementary material for: Development of SAFT-Based Coarse-Grained Models of Carbon Dioxide and Nitrogen
Source: J Phys Chem B. 2025 Mar 21;129(13):3443–53. doi: 10.1021/acs.jpcb.5c00536 (PMC11973872; doi:10.1021/acs.jpcb.5c00536)
Supplement: Supplementary file 1 — jp5c00536_si_001.pdf [file jp5c00536_si_001.pdf]

Supplementary Information: Development of SAFT-based Coarse-Grained Models of Carbon Dioxide and Nitrogen

Alexandros Chremos,[1, a)] William P. Krekelberg,[1] Harold W. Hatch,[1] Daniel W. Siderius,[1] Nathan A. Mahynski,[1] and Vincent K. Shen[1]

Chemical Sciences Division, National Institute of Standards and Technology, Gaithersburg, MD, 20899–8320, USA

(Dated: 3 March 2025)

I. ADDITIONAL DETAILS ON SIMULATION METHODOLOGY

WL-TMMC GC simulations with 0 to $N_{\max}$ molecules as the macrostate were parallelized on 32 processors by separating the macrostate range into subsets of size determined by the exponential parameter 2.25 and reconstructing the collection matrix over the entire macrostate range.[1,2] To speed up the simulations of non-integer λ_r and λ_a, Eq. 1 of the main text was tabulated with 10^4 elements using linear interpolation in the transformed variable $z = (r^{-2} - r_h^{-2})/(r_c^{-2} - r_h^{-2})$, where U below $r_h/\sigma = 0.85$ was infinite and $r_c/\sigma = 3$. Rotations were performed by fixing one segment and rotating the other by a randomly generated quaternion[3] subject to the maximum rotation parameter. The collection matrix was updated after 20 Wang-Landau flatness checks, and transition-matrix was used for the macrostate bias after 25 Wang-Landau flatness checks and one sweep of the collection matrix, where one sweep is when each macrostate has been visited by another macrostate at least 100 times.[4] For each model and temperature, an approximate guess for μ and $N_{\max}$ were chosen to be near the equilibrium condition where the probability of the largest macrostate was less than 10^{-4}. Single-component vapor-liquid equilibrium densities and pressures were obtained by reweighting the macrostate probability distributions.[5,6]

II. SINGLE-SEGMENT MODELING

We briefly discuss our findings by modeling N_2 as a single-segment chain fluid with SAFT-γ Mie; this approach is similar to the development of CO_2 model within the same framework.[8] We performed a parameter estimation for N_2 with Despasito[9] in the temperature range between 60 K and 100 K and used as a point of reference the saturated liquid density and pressure of NIST correlations[7] for N_2 to obtain the optimum values. The resulting parameter set is presented in Table S1. Visual inspection of the performance is presented in Fig. S1. There are no overlap issues in the case of single-segment models, i.e., we have $S = 1$.

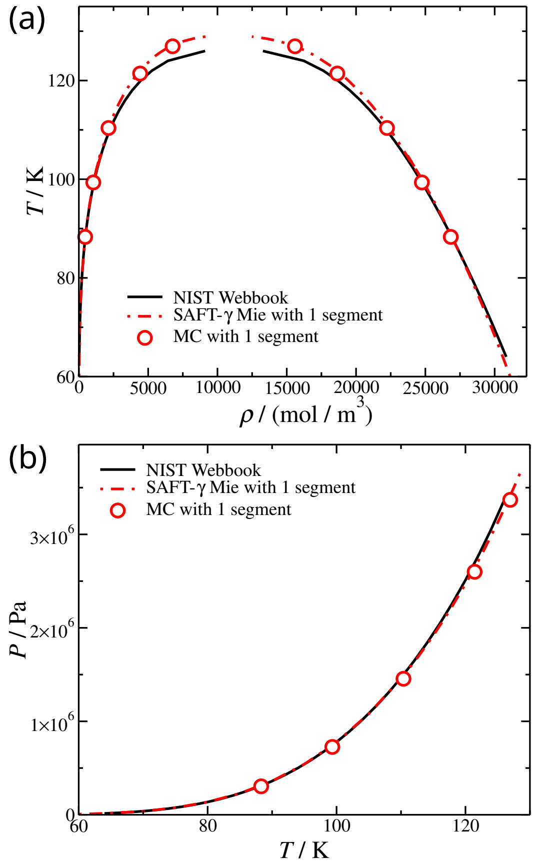

FIG. S1. (a) Coexisting densities $T\rho$ and (b) vapor pressure PT phase diagrams for N_2. The black continuous lines correspond to the NIST correlations for N_2.[7] Open symbols correspond to Monte Carlo simulations with a single segment chain model based on SAFT-γ Mie parameters, see Table S1.

This means that the parameter sets for molecular models composed of a single segment chain fluid obtained in SAFT-γ Mie can used in molecular simulations without any adjustments. The SAFT predictions and the molecular simulations provided a good description of N_2 over a wide range of temperatures and pressures. The performance of single-segment CO_2 is discussed in Ref. 8.

We performed a parameter optimization on the phase behavior of the CO_2+N_2 mixture at $T = 233$ K to determine the cross-energy interaction; the mixing rules determined all other parameters, see Eqns. 3, 4, & 5 of the main paper. The cross-interaction parameters are

[a)] Electronic mail: alexandros.chremos@nist.gov

TABLE S1. SAFT-γ Mie self- and cross-interaction parameters for the functional groups for CO_2 and N_2. These parameter sets are based on single-segment chain fluid models.

Group(s)	n	S	σ/Å	(ε/k_B)/K	λ_r	λ_a	Ref.
CO_2	1	1.0	3.741	361.69	23.0	6.66	8
N_2	1	1.0	3.633	110.383	15.214	5.948	†
CO_2+N_2	-	-	3.685	205.232	16.892	6.358	†

† Current work.

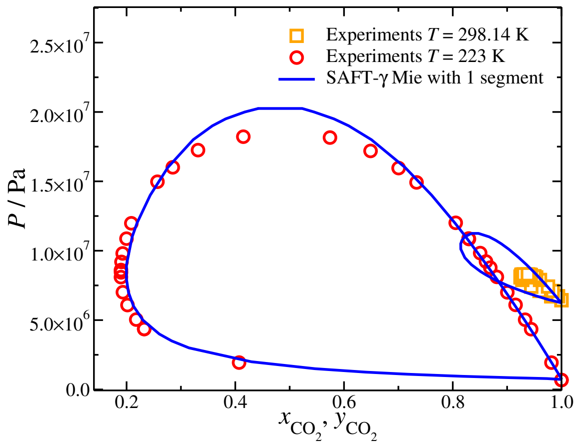

FIG. S2. Isothermal pressure-composition slices of the vapor-liquid equilibrium of N_2 + CO_2 binary mixture. The continuous lines represent the SAFT-γ Mie predictions based on the parameter set in Table S1. Open symbols represent experimental bubble and dew point pressures.[10]

presented in Table S1. We found very good agreement at temperatures away from the critical point of CO_2. However, we found deviations growing as the temperature was increased towards the critical temperature of CO_2, see Fig. S2. While this is expected near critical points, the deviation was larger and occurred at temperatures lower than expected. Additional optimizations were performed, where we relaxed other molecular parameters to improve the phase behavior description at elevated temperatures (results not shown here). While our effort was not exhaustive, we did not find an optimization pathway to considerably improve the predictions of the phase behavior of binary mixtures with single-segment models. A potential solution would be to have temperature-dependent cross-interaction parameters, which would be part of a future investigation. These results suggest that molecular anisotropy may play a significant role in the VLE description and, in particular, in the phase behavior of mixtures. We did not evaluate the resulting simulation models in binary mixtures based on these findings. Instead, we focused on utilizing the two-segment chain fluid description for

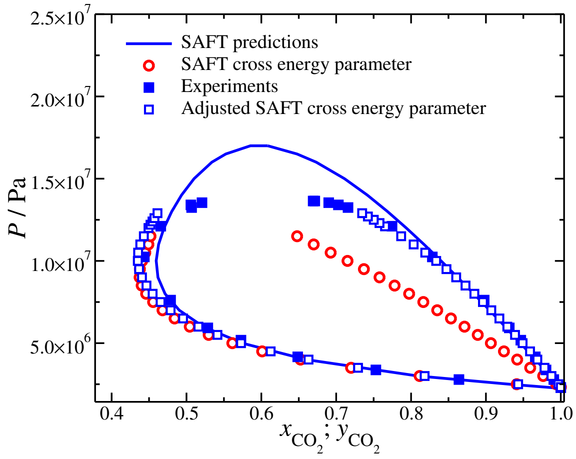

FIG. S3. Isothermal pressure-composition slices of the vapor-liquid equilibrium of N_2 + CO_2 binary mixture. The continuous line represents the SAFT-γ Mie predictions based on the parameter set in Table I in the main paper. Filled symbols represent experimental bubble and dew point pressures by Fadiño *et al.*.[11] Open symbols correspond to MC simulations based on the CG parameter set in Table I in the paper with squares as is, i.e., the cross-energy parameter (ε/k_B)/K = 121.500, and circles corresponding to the same parameter set except that the cross-energy parameter, (ε/k_B)/K = 116.7993, which corresponds to the value obtained from the mixing rules based on SAFT parameters.

CO_2 and N_2, as described in the main paper.

III. TWO-SEGMENT MODELING

The cross-energy interaction parameter was adjusted through linear interpolation by minimizing the deviation of the SAFT molecular model in molecular simulation from the experimental phase behavior. To demonstrate the significance of this adjustment, see Fig. S3.

IV. REFERENCES

[1] K. S. Rane, S. Murali, and J. R. Errington, "Monte Carlo Simulation Methods for Computing Liquid–Vapor Saturation Properties of Model Systems," J. Chem. Theory Comput. **9**, 2552–2566 (2013).

[2] H. W. Hatch, D. W. Siderius, J. R. Errington, and V. K. Shen, "Efficiency Comparison of Single- and Multiple-Macrostate Grand Canonical Ensemble Transition-Matrix Monte Carlo Simulations," J. Phys. Chem. B **127**, 3041–3051 (2023).

[3] F. J. Vesely, "Angular Monte Carlo integration using quaternion parameters: a spherical reference potential for CCl4," J. Comput. Phys. **47**, 291–296 (1982).

[4] H. W. Hatch, J. Mittal, and V. K. Shen, "Computational study of trimer self-assembly and fluid phase behavior," J Chem. Phys. **142**, 164901 (2015).

[5] J. R. Errington, "Direct calculation of liquid-vapor phase equilibria from transition matrix Monte Carlo simulation," J. Chem. Phys. **118**, 9915–9925 (2003).

[6] D. W. Siderius, H. W. Hatch, J. R. Errington, and V. K. Shen, "Comments on "monte carlo simulations for water adsorption in porous materials: Best practices and new insights"," AIChE J. **68**, e17686 (2022).

[7] P. J. Linstrom and W. G. Mallard, Eds., "NIST Chemistry WebBook, NIST Standard Reference Database Number 69, National Institute of Standards and Technology, Gaithersburg MD, 20899 (retrieved November 20, 2012)," (2012).

[8] C. Avendaño, T. Lafitte, A. Galindo, C. S. Adjiman, G. Jackson, and E. A. Müller, "SAFT-γ force field for the simulation of molecular fluids. 1. A single-site coarse grained model of carbon dioxide," J. Phys. Chem. B. **115**, 11154–11169 (2011).

[9] J. A. Clark, N. Duff, A. Abi-Mansour, and E. Santiso, "DESPASITO: A Python Package for SAFT EOS Parametrization and Thermodynamic Calculations," JOSS **10**, 7365 (1984).

[10] S. F. Westman, H. J. Stang, S. Løvseth, A. Austegard, I. Snustad, S. Ø. Størset, and I. S. Ertesvåg, "Vapor–liquid equilibrium data for the carbon dioxide and nitrogen (CO_2+ N_2) system at the temperatures 223, 270, 298 and 303 k and pressures up to 18 mpa," Fluid Phase Equilib. **409**, 207–241 (2016).

[11] O. Fandiño, J. P. M. Trusler, and D. Vega-Maza, "Phase behavior of (CO_2 + H_2) and (CO_2 + N_2) at temperatures between (218.15 and 303.15) K at pressures up to 15 MPa," Int. J. Greenh. Gas Control **36**, 78–92 (2015).
